# Association between *matrix metalloproteinase-3* gene polymorphisms and tendon-ligament injuries: evidence from a meta-analysis

**DOI:** 10.1186/s13102-022-00421-5

**Published:** 2022-02-16

**Authors:** Rui Guo, Aihaiti Aizezi, Yong Fan, Zhe Ji, Wenzong Li, Yongxian Li, Zhigang Wang, Kai Ning

**Affiliations:** 1 Department of Orthopedic Center, People’s Hospital of Xinjiang Uygur Autonomous Region, No. 91 Tianchi Road, Urumqi, 830001 Xinjiang China; 2 Department of Surgery, People’s Hospital of Burqin County, Altay, 836500 Xinjiang China; 3 Department of Surgery, People’s Hospital of Tuoli County, Tacheng, 834300 Xinjiang China

**Keywords:** *Matrix metalloproteinase*-*3*, Polymorphism, Tendon-ligament injury, Meta-analysis

## Abstract

**Background:**

Tendon-ligament injuries (TLIs), including Achilles tendinopathy, cruciate ligament injury, tennis elbow, rotator cuff injury, patellar tendinopathy, and tibial tendinopathy, are common musculoskeletal soft injuries during physical activity. *Matrix metalloproteinase-3* (*MMP-3*) gene polymorphisms have been implicated in the etiology of TLIs in several genetic association studies with inconsistent results. The purpose of this study was to collect and synthesize the current evidences on the association of *MMP-3* polymorphisms and TLIs.

**Methods:**

The search was conducted using PubMed, Web of Science, EMBASE, Cochrane Library, CNKI and Wanfang databases, prior to July, 2021. Newcastle Ottawa Scale was used to appraise the study quality. Strengths of association were represented by odds ratios (ORs) and 95% confidence intervals (95% CIs).

**Results:**

Thirteen studies with 2871 cases and 4497 controls met the eligibility criteria, and each study was in high quality. The overall analyzes suggested *rs3025058* was associated with an increased TLIs risk (5A vs. 6A, OR = 1.20, 95% CI 1.03–1.40, *P* = 0.020). However, the association was not found for *rs679620*, *rs591058*, and *rs650108* polymorphisms. Subgroup analysis by injury type suggested that *rs679620* polymorphism was associated with a reduced risk to Achilles tendon rupture (AA + AG vs. GG, OR = 0.46, 95% CI 0.25–0.87, *P* = 0.020), and *rs3025058* was associated with an elevated risk to anterior cruciate ligament injury (5A5A + 5A6A vs. 6A6A, OR = 1.46, 95% CI 1.03–2.06, *P* = 0.030). When stratified by ethnicity, the findings indicated that *rs3025058* polymorphism was associated with an increased TLIs risk among Caucasians (5A6A vs. 6A6A, OR = 1.55, 95% CI 1.09–2.42, *P* = 0.020) and Brazilians (5A5A vs. 5A6A + 6A6A, OR = 2.80, 95% CI 1.44–5.45, *P* = 0.002).

**Conclusion:**

Findings of this study suggest that *rs679620* polymorphism is associated with a reduced Achilles tendon rupture risk, and *rs3025058* polymorphism contributes to an increased TLIs risk in Caucasians and Brazilians. However, *rs591058* and *rs650108* polymorphisms do not show any association with TLIs.

## Background

Tendons and ligaments within the upper and lower limbs, such as anterior cruciate ligament, rotator cuff tendon, patellar tendon, and Achilles tendon, are common sites of musculoskeletal soft tissue injuries during participating in physical activity [1]. It has been reported that the lifetime prevalence of tendon injuries was up to 23.9% among athletes and 5.9% in the general population [2]. Ligament injuries also occur in millions of individuals [3, 4]. A considerable number of these affected individuals require surgery, imposing a heavy burden on society.

Both of tendons and ligaments are dense connective tissues which are composed of mesenchymal-derived cells [5]. Despite differing in anatomical locations and function, the two tissues are similar in basic components and molecular features [5]. Hence, it is conceivable that tendon-ligament injuries (TLIs) may share similar biological mechanisms.

Despite both extrinsic and intrinsic factors were identified predisposing to TLIs, the exact etiology of these injuries remains largely uncharacterized [6]. Genetic factors are suggested as intrinsic factors for TLIs. Familial studies have provided evidences that genetics may contribute to TLIs [7, 8]. In recent years, high-throughput sequencing technique has resulted in substantial advance in the establishment of association between genetic markers and diseases. Genes like *type I collagen alpha1* (*COL1A1*) [9, 10], *type V collagen alpha1* (*COL5A1*) [11, 12], *fibrillin-2* [13], *matrix metalloproteinase-1* (*MMP-1*) [14, 15], and *MMP-3* [16, 17] have been reported to be associated with TLIs. *MMP-3* gene is one of the most extensively studied candidate genes.

MMP-3 protein, also known as transin/stromelysin, belongs to matrix metalloproteinases (MMPs) which are a cluster of zinc-dependent endopeptidases participating in the breakdown of extracellular matrix [18]. MMP-3 is encoded by *MMP-3* gene, which is mapped to human chromosome 11q22.3. This endopeptidase possesses the function of degrading multiple substrates including fibronectin, laminin, collagens, and cartilage proteoglycans [19].

Raleigh et al. [20] initially reported that the GG genotype of *rs679620* in *MMP3* gene contributed to an increased risk to Achilles tendinopathy in Caucasians. Thus far, a number of studies have looked into *MMP-3* polymorphisms and their impact on susceptibility to TLIs, with varying and inconclusive results. Some studies found that *MMP-3* polymorphisms were significantly associated with TLIs [16, 17, 21, 22], while other studies obtained a null association [23, 24]. The inconformity may be caused by limited sample sizes, population stratification, clinical heterogeneity, and other factors. Therefore, the current evidence-based study was performed to gain a better understanding of the association of *MMP-3* polymorphisms and susceptibility to several common TLIs, including tendon injury, cruciate ligament injury, tennis elbow, rotator cuff injury, and so forth.

## Materials and methods

### Literature search

Literature search of this meta-analysis was from six databases including PubMed, Web of Science, EMBASE, Cochrane Library, China National Knowledge Infrastructure (CNKI), and Wanfang. All studies were published prior to July, 2021. No language restriction was set. The search strategy for PubMed database was: (*Matrix metalloproteinase-3* OR *MMP-3* OR *MMP 3* OR *Metalloproteinase 3, Matrix* OR *Transin* OR *MMP3 Metalloproteinase* OR *Stromelysin 1* OR *Stromelysin*) AND (SNP OR Mutation OR Variant OR Variation OR Polymorphism) and (Achilles tendon OR Tendon injury OR Achilles tendon pathology OR Achilles tendinopathy OR Achilles tendon rupture OR ACL injury OR Anterior cruciate ligament injury OR Ligament injury OR Anterior cruciate ligament tear OR ACL tear OR Tennis elbow OR Lateral epicondylitis OR Rotator cuff tear). This search strategy was transferred into corresponding search strategies in other databases. References in relevant reviews and full-text articles were screened to retrieve additional studies.

### Inclusion and exclusion criteria

Studies were enrolled based upon the following criteria: (1) Comparative studies concerning the association of *MMP-3* gene polymorphisms and TLIs; (2) TLIs were confirmed by clinical and/or imaging criteria; (3) Data on genotype frequency were reported to evaluate odds ratios (ORs) with 95% confidence intervals (95%CIs).

Correspondingly, the excluded criteria were: (1) The study did not satisfy the inclusion criteria; (2) Conference paper, review article, animal study, and case series; (3) Duplicate studies. If data were reported for more than once, the most comprehensive one was selected.

### Data extraction

Two investigators (RG and AA), independently of each other, performed the data extraction according to a standardized form. The data extracted were: author's name, publication year, country or region, ethnicity, study design, detailed genotype frequency of cases and controls, and Hardy–Weinberg Equilibrium (HWE). A study with more than one independent cohort should be separated into several individual studies. In the event of any discrepancy, two investigators double-checked the articles together and resolved the discrepancy by discussion.

### Quality assessment

Two investigators (RG and AA) respectively appraised the quality of eligible studies by using Newcastle Ottawa Scale (NOS) [25]. The NOS included three sections: selection, comparability, and exposure. For the “selection” and “exposure” categories, one point could be awarded for each item. For the “comparability” category, a maximum of two points could be awarded. A study was considered to be in high quality with ≥ 6 scores. Disagreements between two investigators were resolved by discussion.

### Statistical analysis

It was assumed that “V” was the variant allele, “W” was the wild allele, the genotypes of case and control subjects could be grouped into three types including VV, VW, and WW. In the current meta-analysis, five genetic models were investigated, including allele model (V vs. W), homozygous model (VV vs. WW), heterozygous model (VW vs. WW), dominant model (VV + VW vs. WW), and recessive model (VV vs. VW + WW). The strengths of the association were represented by using ORs and 95% CIs. The effect sizes were described as significant if *P* values < 0.05. The heterogeneity between studies was determined using Q-statistical test and *I*^2^ test. When considerable heterogeneity was observed (*P* < 0.10 and *I*^2^ > 50%), the effect size was combined with the random-effects model; otherwise, the fixed-effects model was employed. Subgroup-analysis by injury type and ethnicity were performed.

### Sensitivity analysis and publication bias

Sensitivity analysis was performed by sequentially excluding each individual study to appraise their influence on the ORs and 95% CIs. Publication bias was examined by visual inspection of the symmetry of the funnel plots (the more symmetrical, the lower risk of publication bias). All statistical analyses were carried out using RevMan 5.3 software.

## Results

### Literature search

**Fig. 1:**
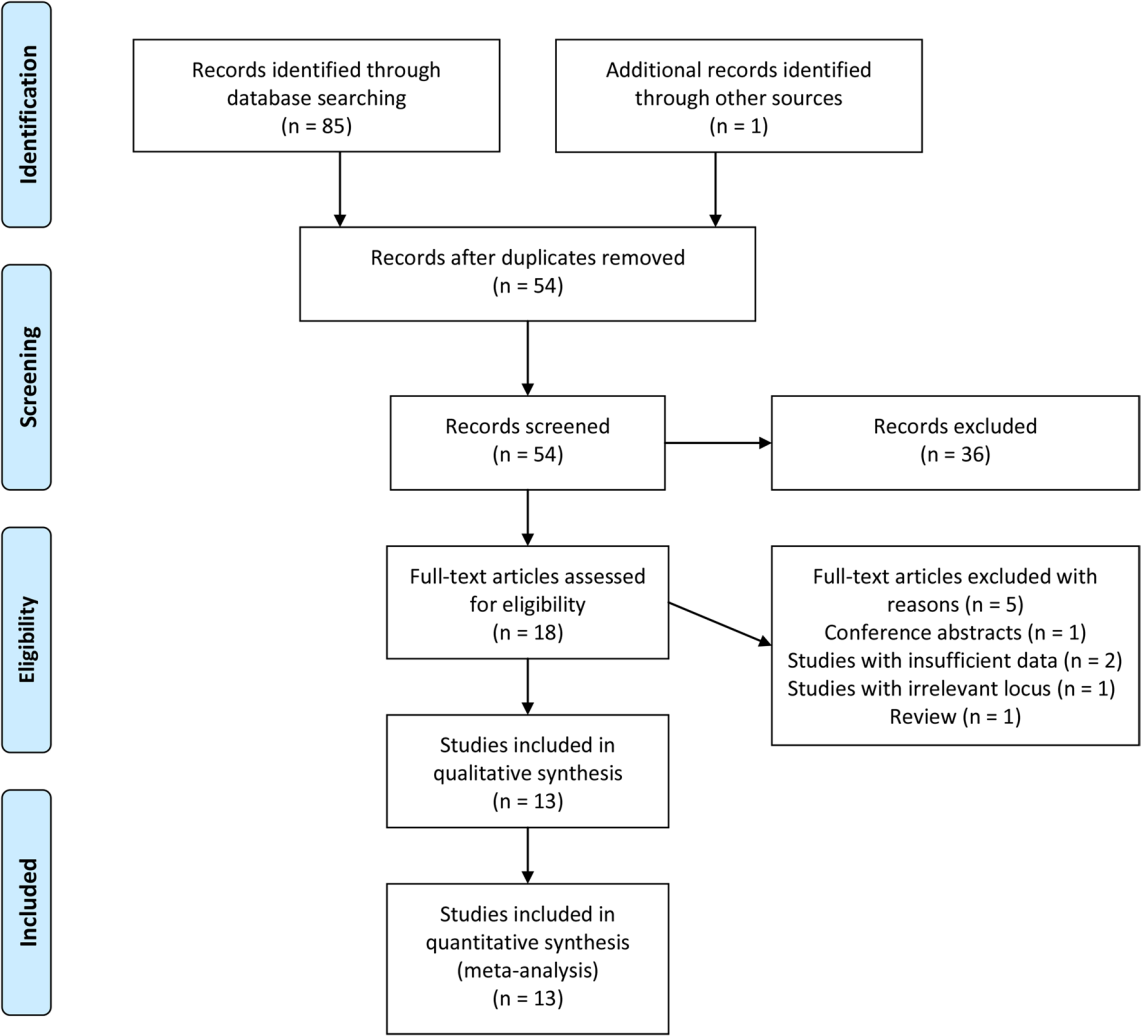
Flow diagram of study identification and selection based upon the inclusion and exclusion criteria

After the search of six databases, 86 potentially relevant items were identified, including 24 from PubMed, 25 from EMBASE, 29 from Web of Science, zero from Cochrane Library, one from Wanfang, six from CNKI, and one from other sources. Thirty-two duplicated records were removed after the initial screen. A further 36 records were excluded after screening the titles and abstracts. Of the remaining 18 items requiring full-text review, another five items were excluded with reasons (two records with insufficient data, one review, one record with irrelevant locus, and one conference abstract). Ultimately, thirteen original studies [16, 17, 20–24, 26–31] fell within the scope of this meta-analysis. The flow diagram was presented in Fig. 1.

### Study characteristics and quality assessment

Total participants, including 2871 cases and 4497 controls, could be grouped into Asians, Caucasians, and mixed population (Brazilians). All studies were published in English, and the publish year ranged from 2009 to 2020. Injury types in the original studies including rotator cuff tear (RCT) [17, 24, 26], Achilles tendinopathy (ATEN) [16, 20, 21, 27, 28], anterior cruciate ligament rupture (ACLI) [22, 23, 28, 31], Achilles tendon rupture (ARUP) [20, 27], patellar tendinopathy (PTEN) [30], and tibial tendinopathy (TTEN) [14]. All the included studies were case–control studies, with the exception of Haug et al.’s [30] cohort study. Except for the studies conducted by Assunção et al. [26] and Godoy-Santos et al. [14] on *rs3025058* polymorphism, all the studies complied with HWE. Of note, the study by El Khoury et al. [27], Raleigh et al. [20], and Gibbon et al. [28] contained two, two, and three different case–control cohorts, respectively. The characteristics of each study were summarized in Table 1. According to NOS, all studies received ≥ 6 scores, labeled as excellent quality (Table 2).

**Table 1:** Main characteristics of included studies

Author	Year	Country/region	Ethnicity	Design	Diagnosis	Case			Control			HWE
						VV	VW	WW	VV	VW	WW	
*Rs679620*						AA	AG	GG	AA	AG	GG	
Briški N	2020	Croatia	Caucasian	Case–control	ATEN	16	30	17	9	51	32	0.08
El Khoury L (I)	2016	UK	Caucasian	Case–control	ATEN	31	46	16	35	71	25	0.31
El Khoury L (II)	2016	UK	Caucasian	Case–control	ARUP	6	8	11	35	71	25	0.31
Figueiredo EA	2020	Brazil	Mixed	Case–control	RCT	41	111	58	90	275	200	0.78
Gibbon A (I)	2017	Australia	Caucasian	Case–control	ATEN	20	40	17	39	94	58	0.94
Haug KBF	2018	Norway	Caucasian	Cohort	PTEN	5	21	7	22	53	18	0.18
Lulinska-Kuklik E	2019	Poland	Caucasian	Case–control	ACLI	54	107	68	59	93	40	0.77
Nie G	2019	China	Asian	Case–control	ATEN	78	460	546	234	950	1004	0.68
Posthumus M	2012	South Africa	Caucasian	Case–control	ACLI	53	94	34	57	99	59	0.25
Raleigh SM (I)	2009	South Africa	Caucasian	Case–control	ATEN	15	32	28	24	55	19	0.22
Raleigh SM (II)	2009	South Africa	Caucasian	Case–control	ARUP	8	20	10	24	55	19	0.22
*Rs591058*						TT	TC	CC	TT	TC	CC	
Briški N	2020	Croatia	Caucasian	Case–control	ATEN	10	25	13	9	51	32	0.08
Gibbon A (I)	2017	Australia	Caucasian	Case–control	ATEN	20	39	18	38	93	60	0.86
Gibbon A (III)	2017	South Africa	Caucasian	Case–control	ACLI	80	182	64	59	106	60	0.39
Haug KBF	2018	Norway	Caucasian	Cohort	PTEN	5	21	7	22	51	20	0.35
Lulinska-Kuklik E	2019	Poland	Caucasian	Case–control	ACLI	54	107	68	59	93	40	0.77
Raleigh SM (I)	2009	South Africa	Caucasian	Case–control	ATEN	15	32	26	25	53	19	0.34
Raleigh SM (II)	2009	South Africa	Caucasian	Case–control	ARUP	9	20	10	25	53	19	0.34
*Rs650108*						GG	GA	AA	GG	GA	AA	
Briški N	2020	Croatia	Caucasian	Case–control	ATEN	35	23	5	31	50	11	0.18
Gibbon A (I)	2017	Australia	Caucasian	Case–control	ATEN	34	27	6	90	56	13	0.32
Gibbon A (III)	2017	South Africa	Caucasian	Case–control	ACLI	205	99	15	122	90	12	0.38
Haug KBF	2018	Norway	Caucasian	Cohort	PTEN	19	13	1	54	35	4	0.58
Raleigh SM (I)	2009	South Africa	Caucasian	Case–control	ATEN	39	28	7	51	42	2	0.05
Raleigh SM (II)	2009	South Africa	Caucasian	Case–control	ARUP	20	16	2	51	42	2	0.05
*Rs3025058*						5A5A	5A6A	6A6A	5A5A	5A6A	6A6A	
Assunção JH	2017	Brazil	Mixed	Case–control	RCT	15	38	11	4	44	16	0.01
Gibbon A (I)	2017	Australia	Caucasian	Case–control	ATEN	20	40	17	40	93	57	0.86
Gibbon A (II)	2017	South Africa	Caucasian	Case–control	ATEN	12	30	27	24	51	18	0.33
Gibbon A (III)	2017	South Africa	Caucasian	Case–control	ACLI	77	185	64	57	108	60	0.55
Godoy-Santos AL	2017	Brazil	Mixed	Case–control	TTEN	15	34	19	12	61	27	0.02
Malila S	2011	Thailand	Asian	Case–control	ACLI	1	22	63	1	20	79	0.84
Miao K	2019	China	Asian	Case–control	RCT	6	48	96	8	42	100	0.21

*V* variant allele, *W* wild allele, *HWE* Hardy–Weinberg Equilibrium, *ATEN* achilles tendinopathy, *ARUP* achilles tendon rupture, *ACLI* anterior cruciate ligament injury, *PTEN* patellar tendinopathy, *RCT* rotator cuff tear, *TTEN* tibial tendinopathy

**Table 2:** Quality assessment of included studies

Study ID	Selection				Control for important factor	Exposure			
	Adequate definition of cases	Representativeness of cases	Selection of control subjects	Definition of control subjects		Exposure assessment	Same method of ascertainment for all subjects	Non-response rate	Total
Assunção JH, 2017	★	☆	★	★	★★	★	★	★	8
Briški N, 2020	★	☆	☆	★	★☆	★	★	★	7
El Khoury L, 2016	★	☆	★	★	★★	★	★	★	8
Figueiredo EA, 2020	★	☆	☆	★	★★	★	★	★	7
Gibbon A, 2017	★	☆	★	★	★☆	★	★	★	7
Haug KBF, 2018	★	★	★	★	★☆	★	★	★	8
Lulinska-Kuklik E, 2019	★	★	★	★	★☆	★	★	★	8
Malila S, 2011	★	☆	☆	★	★★	★	★	★	7
Miao K, 2019	★	☆	★	★	★☆	★	★	★	7
Nie G, 2019	★	☆	★	★	★★	★	★	★	8
Posthumus M, 2012	★	☆	★	★	★☆	★	★	★	7
Raleigh SM, 2009	★	☆	★	★	★☆	★	★	★	7

### Meta-analysis and subgroup analysis

The strengths of the association of *rs679620*, *rs591058*, *rs650108*, and *rs3025058* polymorphisms and TLIs risk were displayed in Table 3.

**Table 3:** Associations of *matrix metalloproteinase 3* gene polymorphisms and tendon-ligament injuries

Genetic model	Test of association			No. of cohorts	Test of association		Statistical model
	OR	95%CI	*P*		I^2^ (%)	*P*	
*Rs679620*							
A versus G							
Overall	0.97	0.81–1.17	0.780	11	72	< 0.001	R
Caucasian	0.96	0.76–1.21	0.740	9	66	0.003	R
ATEN	1.04	0.77–1.39	0.810	5	76	0.002	R
ARUP	0.70	0.47–1.04	0.080	2	0	0.400	F
ACLI	0.95	0.56–1.64	0.860	2	87	0.006	R
AA versus GG							
Overall	0.95	0.64–1.40	0.790	11	73	< 0.001	R
Caucasian	0.94	0.58–1.52	0.810	9	66	0.002	R
ATEN	1.10	0.56–2.14	0.790	5	79	< 0.001	R
ARUP	0.50	0.23–1.09	0.080	2	0	0.550	F
ACLI	0.93	0.32–2.72	0.890	2	87	0.006	R
AG versus GG							
Overall	0.92	0.71–1.20	0.540	11	61	0.004	R
Caucasian	0.84	0.57–1.23	0.370	9	61	0.009	R
ATEN	0.89	0.78–1.03	0.120	5	46	0.120	F
ARUP	0.45	0.23–0.87	0.020	2	50	0.160	F
ACLI	1.05	0.44–2.52	0.910	2	84	0.010	R
AA + AG versus GG							
Overall	0.92	0.70–1.22	0.580	11	70	< 0.001	R
Caucasian	0.86	0.58–1.28	0.460	9	68	0.002	R
ATEN	0.95	0.65–1.40	0.800	5	63	0.030	R
ARUP	0.46	0.25–0.87	0.020	2	37	0.210	F
ACLI	1.01	0.39–2.59	0.990	2	88	0.004	R
AA versus AG + GG							
Overall	1.00	0.77–1.30	1.000	11	57	0.009	R
Caucasian	1.03	0.83–1.26	0.810	9	40	0.100	F
ATEN	0.85	0.70–1.05	0.130	5	77	0.002	R
ARUP	0.84	0.43–1.65	0.610	2	0	0.940	F
ACLI	0.89	0.55–1.46	0.650	2	60	0.110	R
*Rs591058*							
T versus C							
Overall	0.96	0.76–1.20	0.700	7	59	0.020	R
ATEN	1.08	0.66–1.77	0.760	3	75	0.020	R
ACLI	0.90	0.59–1.37	0.630	2	81	0.020	R
TT versus CC							
Overall	0.93	0.58–1.51	0.780	7	61	0.020	R
ATEN	1.25	0.43–3.59	0.680	3	76	0.020	R
ACLI	0.83	0.36–1.93	0.670	2	81	0.020	R
TC versus CC							
Overall	0.97	0.66–1.42	0.860	7	57	0.030	R
ATEN	0.91	0.44–1.8	0.800	3	66	0.050	R
ACLI	1.05	0.45–2.46	0.910	2	86	0.008	R
TT + TC versus CC							
Overall	0.95	0.66–1.41	0.800	7	64	0.010	R
ATEN	0.98	0.45–2.17	0.970	3	75	0.020	R
ACLI	0.97	0.41–2.27	0.940	2	88	0.005	R
TT versus TC + CC							
Overall	0.90	0.72–1.13	0.370	7	28	0.220	F
ATEN	1.24	0.82–1.90	0.310	3	48	0.150	F
ACLI	0.81	0.61–1.08	0.150	2	0	0.360	F
*Rs650108*							
G versus A							
Overall	1.15	0.96–1.37	0.140	6	44	0.110	F
ATEN	1.06	0.81–1.39	0.670	3	70	0.040	R
GG versus AA							
Overall	1.03	0.64–1.67	0.900	6	31	0.200	F
ATEN	0.85	0.24–2.92	0.790	3	66	0.050	R
GA versus AA							
Overall	0.77	0.47–1.26	0.300	6	0	0.520	F
TEN	0.71	0.36–1.39	0.320	3	40	0.190	F
GG + GA versus AA							
Overall	0.91	0.57–1.46	0.700	6	8	0.360	F
ATEN	0.77	0.28–2.13	0.610	3	53	0.120	R
GG versus GA + AA							
Overall	1.26	1.00–1.57	0.050	6	45	0.100	F
ATEN	1.21	0.62–2.36	0.570	3	72	0.030	R
*Rs3025058*							
5A versus 6A							
Overall	1.20	1.03–1.40	0.020	6	0	0.740	F
Caucasian	1.16	0.95–1.42	0.510	2	0	0.510	F
Brazilians	1.38	1.00–1.92	0.050	2	0	0.340	F
Asian	1.12	0.80–1.56	0.520	2	0	0.540	F
ACLI	1.14	0.91–1.42	0.260	2	0	0.630	F
RCT	1.25	0.92–1.71	0.150	2	50	0.160	F
5A5A versus 6A6A							
Overall	1.48	1.06–2.08	0.020	6	0	0.350	F
Caucasian	1.37	0.91–2.08	0.130	2	0	0.540	F
Brazilians	2.64	1.23–5.67	0.010	2	0	0.180	F
Asian	0.83	0.30–2.30	0.720	2	0	0.760	F
ACLI	1.27	0.78–2.05	0.340	2	0	0.990	F
RCT	1.98	0.30–13.30	0.480	2	79	0.030	R
5A6A versus 6A6A							
Overall	1.31	1.03–1.67	0.030	6	0	0.700	F
Caucasian	1.55	1.09–2.42	0.020	2	0	0.790	F
Brazilians	1.95	0.55–1.67	0.870	2	0	0.430	F
Asian	1.25	0.84–1.88	0.280	2	0	0.730	F
ACLI	1.54	1.07–2.21	0.020	2	0	0.710	F
RCT	1.21	0.78–1.86	0.400	2	0	0.920	F
5A5A + 5A6A versus 6A6A							
Overall	1.32	1.05–1.67	0.020	6	0	0.850	F
Caucasian	1.50	1.07–2.10	0.020	2	0	0.970	F
Brazilians	1.17	0.68–2.00	0.570	2	0	0.350	F
Asian	1.20	0.81–1.77	0.360	2	0	0.640	F
ACLI	1.46	1.03–2.06	0.030	2	0	0.840	F
RCT	1.22	0.81–1.85	0.340	2	0	0.480	F
5A5A versus 5A6A + 6A6A							
Overall	1.36	0.85–2.19	0.200	6	47	0.090	R
Caucasian	1.01	0.72–1.41	0.940	2	0	0.330	F
Brazilians	2.80	1.44–5.45	0.002	2	16	0.280	F
Asian	0.78	0.29–2.15	0.640	2	0	0.770	F
ACLI	0.92	0.62–1.35	0.660	2	0	0.860	F
RCT	1.82	0.30–10.93	0.510	2	80	0.020	R

*OR* odds ratio, *CI* confidence interval, *F* fixed-effects model, *R* random-effects model, *ATEN* Achilles tendinopathy, *ARUP* achilles tendon rupture, *ACLI* anterior cruciate ligament injury, *PTEN* patellar tendinopathy, *RCT* rotator cuff tear, *TTEN* tibial tendinopathy

#### *Rs679620* polymorphism and TLIs

**Fig. 2:**
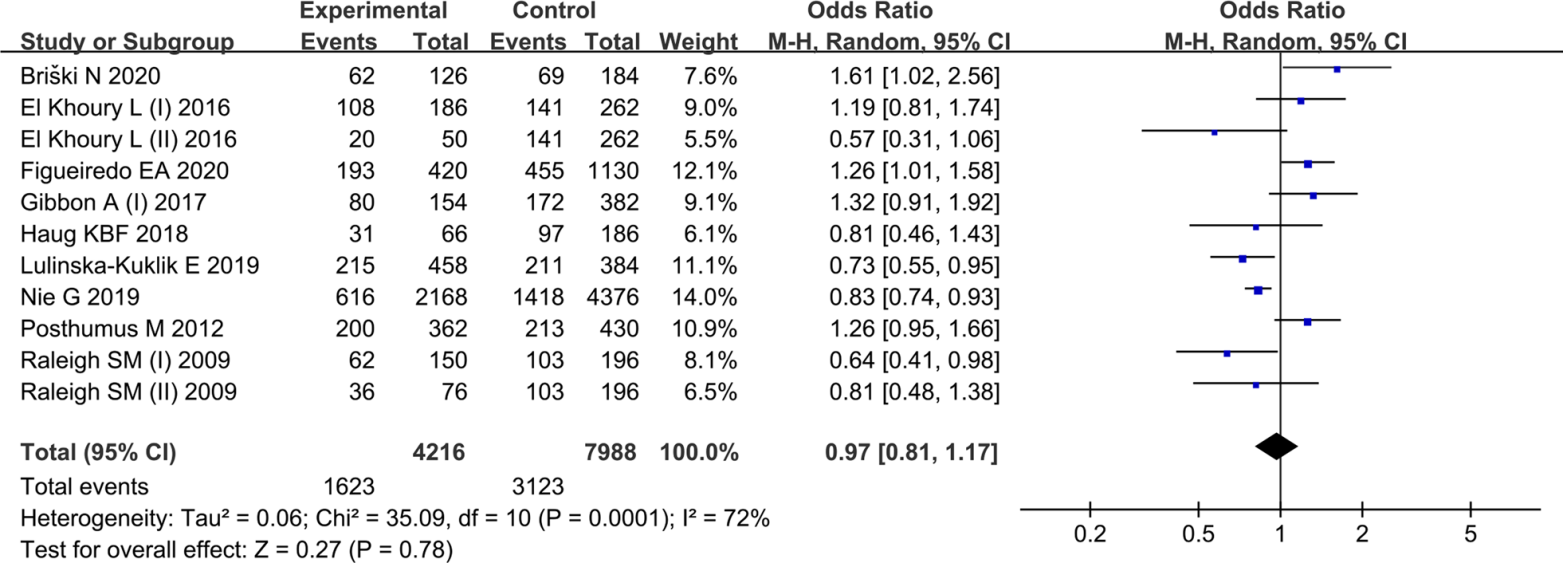
Association between *rs679620* polymorphism and tendon-ligament injuries (A vs. G) in overall populations. The squares and horizontal lines correspond to OR and 95% CI of an individual study. The area of the squares reflects the study weight. Diamond represents the pooled OR and 95% CI. *OR* odds ratio, *CI* confidence interval, *M–H* Mantel–Haenszel method, *Events* the count of A-allele, *Total* the count of A-allele and G-allele

Nine studies [16, 17, 20–23, 27, 28, 30] with 11 cohorts investigated the *rs679620* polymorphism and TLIs vulnerability, encompassing 2108 cases and 3896 controls. Significant between-study heterogeneity was examined in most contrasts. The combined data indicated that *rs679620* polymorphism did not associate with TLIs under any genetic model (Fig. 2). Subgroup analysis by ethnicity suggested *rs679620* was not associated with TLIs in Caucasians. As only one study was conducted in Asians and Brazilians, subgroup analyses for these populations were not conducted. When stratified by injury type, it suggested that *rs679620* was associated with a reduced ARUP risk under heterozygous model (AG vs. GG, OR = 0.45, 95% CI 0.23–0.87, *P* = 0.020) and dominant model (AA + AG vs. GG, OR = 0.46, 95% CI 0.25–0.87, *P* = 0.020). However, no association was observed for ATEN and ACLI under any model.

#### *Rs591058* polymorphism and TLIs

Five studies [16, 20, 22, 28, 30] with seven cohorts examined the association of *rs591058* and TLIs, including 825 cases and 890 controls. All the participants were Caucasians. Because considerable heterogeneity was detected, the random-effects model was used. The pooled data suggested that *rs591058* was not associated with TLIs in the overall Caucasians. Subgroup analysis by injury type indicated that *rs591058* polymorphism was not associated with ATEN or ACLI.

#### *Rs650108* polymorphism and TLIs

Four studies [16, 20, 28, 30] with six cohorts including 204 cases and 251 controls reported the association of *rs650108* and TLIs. All the participants were Caucasians. For the overall Caucasians, the heterogeneity was not significant, and the fixed-effects model was used. The merged data suggested that *rs650108* was not associated with TLIs. Subgroup analysis by injury type also obtained a null association between *rs650108* polymorphism and ATEN.

#### *Rs3025058* polymorphism and TLIs

Five studies [24, 26, 28, 29, 31] with seven cohorts reported the association of *rs3025058* and TLIs, including 412 cases and 325 controls. Significant heterogeneity was detected. Sensitivity analysis found the heterogeneity was mainly from the South African cohort of Gibbon et al.’s study on ATEN [28], which could reverse the merged results. Therefore, Gibbon et al.’s study was excluded. The combined data indicated *rs3025058* was associated with an elevated TLIs risk under allele model (5A vs. 6A, OR = 1.20, 95% CI 1.03–1.40, *P* = 0.020), homozygous model (5A5A vs. 6A6A, OR = 1.48, 95% CI 1.06–2.08, *P* = 0.020), heterozygous model (5A6A vs. 6A6A, OR = 1.31, 95% CI 1.03–1.67, *P* = 0.030), and dominant model (5A5A + 5A6A vs. 6A6A, OR = 1.32, 95% CI 1.05–1.67, *P* = 0.020) in the overall population.

Subgroup analysis by ethnicity indicated *rs3025058* was associated with an increased TLIs risk in Caucasians and Brazilians. When stratified by injury type, it suggested that *rs3025058* was associated with ACLI under heterozygous model and dominant model.

### Sensitivity analysis and publication bias

**Fig. 3:**
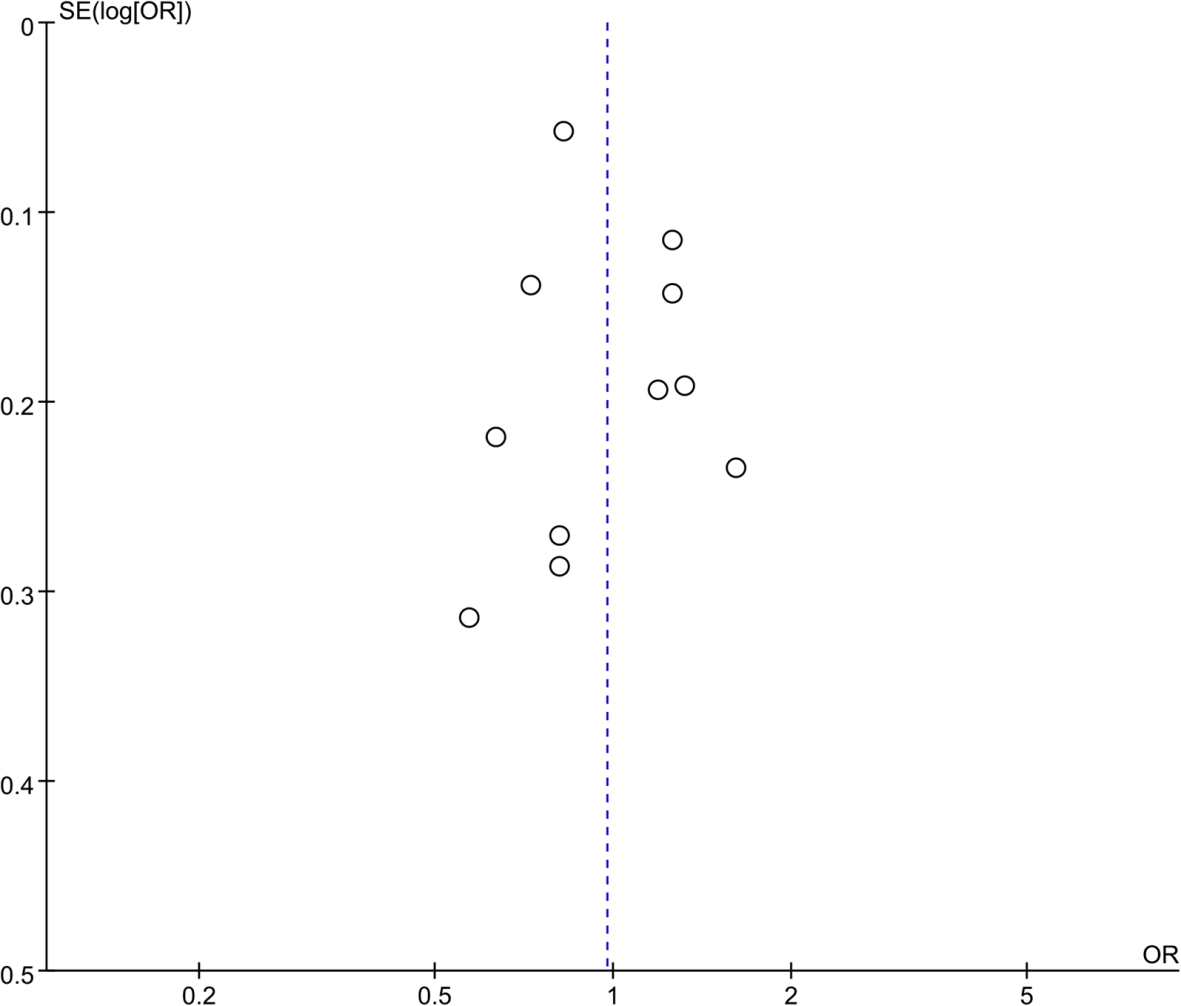
Funnel plot analysis for publication bias of *rs679620* polymorphism and tendon-ligament injuries (A vs. G) in overall populations. The funnel plot appears to be symmetrical, suggesting no significant publication bias

The stableness and robustness of the results were estimated by sensitivity analysis. With sequential removal of an individual study from the analyses, no significant changes were observed in the re-calculated ORs and 95%CIs, which confirmed the reliability of the results. Funnel plots did not exhibit obvious asymmetry, suggesting no significant existence of publication bias (Fig. 3).

## Discussion

This meta-analysis suggested that *rs679620* polymorphism was associated with a reduced ARUP risk, and *rs3025058* polymorphism was associated with an increased TLIs risk in the Caucasians and Brazilians. However, *rs591058* and *rs650108* polymorphisms did not show any association with TLIs. TLIs are common musculoskeletal soft tissue injuries among athletes and physically active individuals. These injuries may prevent the affected individuals from achieving their full potential. Despite TLIs have been widely studied, the underlying mechanisms are still largely indeterminate. A better recognition of the mechanisms of TLIs may assist the prevention, treatment and rehabilitation of these injuries. Risk factors associated with TLIs are traditionally divided into intrinsic and extrinsic factors. Intrinsic risk factors like genetics may predispose individuals to a specific injury.

Polymorphisms within *MMP-3* gene have previously been reported to be associated with several complex musculoskeletal disorders such as osteoarthritis [32], frozen shoulder [33], and disc degeneration [34]. As an intrinsic factor for TLIs, *MMP-3* gene polymorphisms have received a lot of attention in the past years. Genetic association studies often statistically underpowered because of limited sample sizes. Therefore, the existing evidences were combined by a meta-analysis approach to increase the statistical power.

Tendons and ligaments are collagenous tissues with similar composition and structures. MMP-3, also known as stromelysin-1, are involved in the breakdown of collagenous matrix components [18]. It plays an essential role in the remodeling of connective tissues by mediating extracellular matrix homeostasis [35]. It is speculated that an increased expression of *MMP3* gene is of great importance to prevent pathological alterations in tendons [36]. Tendons with tendinopathy appear to have an elevated rate of matrix remodeling, hence making tendons more vulnerable to injury. Similarly, evidence has indicated that the biological activity of MMP-3 is reduced in ruptured tendons [37]. Clinical studies suggested that *MMP-3* mRNA was down-regulated in Achilles tendinopathy tissues compared with control tissues [36, 38]. All these evidences indicate that MMP-3 has an effect on TLIs risk.

*Rs679620* variant is a non-synonymous polymorphism which leads to the substitution of a glutamate residue (GAA) by a lysine residue (AAA) of pro-MMP3. This alteration is speculated to influence the mature of MMP3 enzyme [20]. However, bioinformatics analyses by Nie et al. [21] suggested that this alteration could not significantly alter the biological function of the MMP3 protein. Therefore, the mechanisms underlying *rs679620* polymorphism and TLIs still need to be investigated. In this study, significant association was observed between *rs679620* and ARUP among Caucasians, but the included study number and sample size were quite limited. Further large sample studies are encouraged to verify this association.

*Rs3025058* is featured by the presence of five or six adenines, leading to alleles 5A and 6A. The 5A allele was reported to have an elevated transcriptional activity compared with the 6A allele of *MMP-3* gene [39]. Literature has reported that higher production of MMP-3 in patients with RCT [40]. *Rs591058* polymorphism is a T/C transition at position 1547 situated in intron 4, and *rs650108* polymorphism is a G/A transition at position 495 within intron 8. The functional of the two variants have not yet been established. It is reported that the two polymorphisms are in linkage disequilibrium with other polymorphisms within or beyond *MMP-3* gene and form functional haplotypyes with influences on gene expressions and protein functions [20, 23].

This meta-analysis, despite being performed with a rigorous methodology, has limitations that should be addressed. Firstly, although subgroup analyses and sensitivity analyses were carried out, partial results should be interpreted with caution as a consequence of considerable heterogeneity. Secondly, the vast majority of included studies were retrospective case–control studies, future prospective cohort studies are encouraged to identify the causal association. Third, the cases enrolled in our present study were from different sport groups, which might result in an overestimation or underestimation of the conclusion. Fourth, the collective contribution of several associated polymorphisms and genes was not analyzed because of insufficient data. Last, for *rs3025058* polymorphism, two studies [26, 29] were not in HWE, which might have some influence on the outcome. Therefore, more evidence should be considered from well-designed studies.

## Conclusion

In summary, *rs679620* polymorphism is associated with a reduced ARUP risk, and *rs3025058* polymorphism contributes to an increased TLIs risk in Caucasians and Brazilians. However, *rs591058* and *rs650108* polymorphisms do not show any association with TLIs. Concerning limitations of this study, well-designed prospective cohort studies are encouraged to identify the association of *MMP-3* polymorphisms and TLIs susceptibility.

## Data Availability

The data used in this paper are available from the corresponding author upon reasonable request.
